# A Data Model for Teleconsultation in Managing High-Risk Pregnancies: Design and Preliminary Evaluation

**DOI:** 10.2196/medinform.8393

**Published:** 2017-12-14

**Authors:** Kolsoum Deldar, Fatemeh Tara, Kambiz Bahaadinbeigy, Mohammad Khajedaluee, Mahmood Tara

**Affiliations:** ^1^ Student Research Committee Department of Medical Informatics, Faculty of Medicine Mashhad University of Medical Sciences Mashhad Islamic Republic Of Iran; ^2^ Women Health Research Center Department of Obstetrics and Gynecology, Faculty of Medicine Mashhad University of Medical Sciences Mashhad Islamic Republic Of Iran; ^3^ Medical Informatics Research Center, Institute of Futures Studies in Health Kerman University of Medical Sciences Kerman Islamic Republic Of Iran; ^4^ Department of Community Medicine Faculty of Medicine Mashhad University of Medical Sciences Mashhad Islamic Republic Of Iran; ^5^ Department of Medical Informatics Faculty of Medicine Mashhad University of Medical Sciences Mashhad Islamic Republic Of Iran

**Keywords:** remote consultation, clinical archetype, pregnancy, clinical decision-making

## Abstract

**Background:**

Teleconsultation is a guarantor for virtual supervision of clinical professors on clinical decisions made by medical residents in teaching hospitals. Type, format, volume, and quality of exchanged information have a great influence on the quality of remote clinical decisions or tele-decisions. Thus, it is necessary to develop a reliable and standard model for these clinical relationships.

**Objective:**

The goal of this study was to design and evaluate a data model for teleconsultation in the management of high-risk pregnancies.

**Methods:**

This study was implemented in three phases. In the first phase, a systematic review, a qualitative study, and a Delphi approach were done in selected teaching hospitals. Systematic extraction and localization of diagnostic items to develop the tele-decision clinical archetypes were performed as the second phase. Finally, the developed model was evaluated using predefined consultation scenarios.

**Results:**

Our review study has shown that present medical consultations have no specific structure or template for patient information exchange. Furthermore, there are many challenges in the remote medical decision-making process, and some of them are related to the lack of the mentioned structure. The evaluation phase of our research has shown that data quality (*P*<.001), adequacy (*P*<.001), organization (*P*<.001), confidence (*P*<.001), and convenience (*P*<.001) had more scores in archetype-based consultation scenarios compared with routine-based ones.

**Conclusions:**

Our archetype-based model could acquire better and higher scores in the data quality, adequacy, organization, confidence, and convenience dimensions than ones with routine scenarios. It is probable that the suggested archetype-based teleconsultation model may improve the quality of physician-physician remote medical consultations.

## Introduction

Teleconsultation is an important application of telemedicine, and it could be done remotely between two or more health care providers, for diagnostic or therapeutic purposes, by the use of information and communication technology [1]. In fact, by using this technology, the physician’s knowledge, experience, and services could be used despite the lack of his or her physical presence [2].

### Teleconsultation Models Based on Time

A look at published articles in the field of telemedicine shows that medical consulting can be performed in different ways, especially in four models: real time, near real time, store and forward, and mixed or hybrid. In a real-time model, the physicians in the referral center and refer center are present at the same time for a teleconsultation session. In this model, data and information exchange could be done through methods such as live chat and Internet-based videoconferencing or procedures independent of the Internet, such as a telephone conversation, whereas in the store and forward model, there is no such synchronization between sending patient-related data and receiving the response of the consultant. It is also possible to use Internet-based methods such as email or Web-based forums and non-Internet-based methods such as fax or short message service (SMS) in this model. Some consultations also use methods that are between real-time and asynchronous methods or near real time. In this method, although physicians in both centers are simultaneously in place for giving consultations, there is usually a short delay between sending data and its delivery to the referring center. This method has been used where there is a high volume of data exchange but not enough bandwidth to communicate live on the Web. Examples of this approach include the ability to upload video files from the referral center and receive and view it after a short time in the referring center [3]. Some consultations are also carried out through a combination or mix of the aforementioned models.

### Consultations in Educational Hospitals

In our educational hospitals and in the absence of clinical experts (eg, holidays), the senior and junior residents are responsible for patient care through telephone consulting with an on-call physician. Our previous study had shown that there is no formal education about the consultation process for requesting a resident, and it is learnt only by oral education or observing the performance of senior ones [4]. So, data and information content that is exchanged in these consultations has no standard format or template. On the other hand, type, format, quality, and volume of clinical data exchange is different. These differences can be caused by many factors such as the urgency of clinical conditions of the patient, the Internet speed, experience and background of prior consultations with the applicant physician, receiving or not receiving feedback from the consulting physician, the medium, personality traits of the applicant physician, and time of consultation. Usually clinical findings are summarized by resident, and only prominent points are transferred to an on-call physician. This issue can adversely affect the quality of clinical decisions made by an on-call physician. Additionally, it is important to consider that because of the lack of the consultant’s physical presence beside the patient, changes in the type, quality, and the volume of clinical data provided by the applicant physician can make a significant impact on the quality of the distant physician’s consultation. Physicians’ discontent of poor quality consulting practices and the unstructured data being exchanged has been reported in several studies [5-8]. Moreover, the inadequacy of the data and submitting them as free text are among the problems in the teleconsultation process [9].

### Data Model for Teleconsultation

It might improve the consultation process to use structured templates in the form of a data model for teleconsultation. This means that data items required for decision making of the physician in different conditions are identified. Hence, at the time of consultation, the applicant physician would have already completed and submitted all those necessary items to the consulting physician. On the other hand, to observe the minimum standards for the exchange of data and information, the above items such as templates, formats, and units of measure should be limited.

An effective step on the path to standardizing the exchange of data and information is the use of clinical archetypes. Archetype and template reviews show that some of their main goals of design and deployment are clinical data structuring, increasing interactivity between information systems, preserving data integrity, and simply enhancing quality [10]. However, it seems that such a model has not been used in teleconsultation. If the physicians on either side would use the special tele-decision-making clinical archetype for the data and information exchanging process, it is possible to create better outcomes for patients. As little research has been done to create a standard structure for exchanging data [11], this study was designed and conducted to develop a data model for teleconsultation based on the decision-making archetype for managing high-risk pregnancies.

## Methods

This study was carried out in three phases; the methodology and results of phases 0 and 1 are described in detail in previous studies.

### Phase 0

In this phase, a systematic review was done that aimed to make the physicians familiar with the concept of teleconsultation and finding potential problems. Additionally, a qualitative study was performed to determine the current status of telephone consultations among residents and on-call obstetricians or gynecologists, and a study based on Delphi was carried out to identify high-risk pregnancies in predetermined departments.

### Phase 1

#### Extraction and Localization of Items for Tele-Decision-Making Clinical Archetypes

Main references in obstetrics and gynecology (eg, textbooks, clinical guidelines, and electronic databases) were used to extract the items for tele-decision-making clinical archetypes, and then they were localized in several expert panel sessions. Details are described in a previous study.

#### Designing the Model

After finalizing the “extract and localize” process, the accepted items were divided into distinct subcategories (eg, “amniotic fluid index” was allocated to the “biometric ultrasonography” subcategory). These subcategories were also assigned into larger and more comprehensive groups (eg, the “biometric ultrasonography” subcategory was allocated to the “para-clinic” category). This process continued to achieve the main categories as needed for clinical teleconsultations. Required attributes for each item, such as format acceptable to respond to the items and accepted units of measurement for numeric domains, were defined based on the opinions of clinicians and informatics experts. The model was designed by using Microsoft Visio drawing software.

### Phase 2

The model, which was designed by using pre-prepared consultation scenarios, was evaluated.

#### Designing of Consultation Scenarios to Evaluate the Model

In this phase, one of the qualified volunteers, who was a senior obstetrics and gynecology resident of a teaching hospital of the Mashhad University of Medical Sciences (MUMS), was asked to design five assumptive telephone consulting scenarios in which the physician consulted with an on-call professor based on the items of the tele-decision-making clinical archetypes. These scenarios were designed using five real, paper patient records that described a common high-risk pregnancy (one high-risk pregnancy record for each), so that on the one hand, a list of identified items for the physicians’ tele-decision making and on the other hand, information of patients with hidden identities was placed at his or her disposal to use them to provide assumptive teleconsultation scenarios with professors.

Then, anonymous medical information of the same cases were given to other senior residents who were usually responsible for consulting with on-call professors. They were asked, assuming that their intention was to consult with an on-call physician about the patient by telephone, to provide a typical scenario for every case. A unique code was assigned to each scenario.

#### Designing Checklist

The design of the checklist used in this stage, took place in several phases (described in another study). In short, at the beginning, by using the results of qualitative research (interviews with experts), as well as a broad overview of the texts available in electronic resources and scientific articles, possible items suitable for the design of the checklist were identified. Then, all extracted items were examined and modified by clinicians and informatics experts in three stages to design a checklist tailored to the needs of research that was focused on the teleconsultations.

#### Comparing Routinely Designed Scenarios With an Archetype-Based Scenario

Scenario comparison was developed based on the archetype, with scenarios designed routinely, and these scenarios were randomly placed at the discretion of the clinicians. After reading each consultation scenario, experts commented on the content of consultations and registered them in the predesigned checklist. The allocation of archetype-based scenarios or routine-based scenarios to experts took place randomly. Wilcoxon test was used to compare the scores.

## Results

### Phase 0

#### The Result of Systematic Review

The most important finding of this study is that there is presently no structured format for data and information exchange in teleconsultations [11].

#### The Result of the Qualitative Study

The qualitative study has shown that specialists during teleconsultation with residents faced significant challenges at the time of the diagnosis and treatment of patients; the majority of these problems were caused by insufficient confidence in the judgment of the resident or disproportionate volume of information received concerning the patients [4].

#### The Result of the Delphi Study

Results of this study have revealed that the most common high-risk pregnancies in hospitals in Mashhad are as follows: pre-eclampsia or eclampsia, hemorrhage in the third trimester, postterm delivery, preterm birth, and premature rupture of membranes [12].

### Phase 1

#### The Result of Extraction and Localization of Items for Tele-Decision-Making Clinical Archetypes

This step led to the formation of two groups: general and demographic information (16 items) and technical information (142 items) [12].

#### The Designed Model

**Figure 1:**
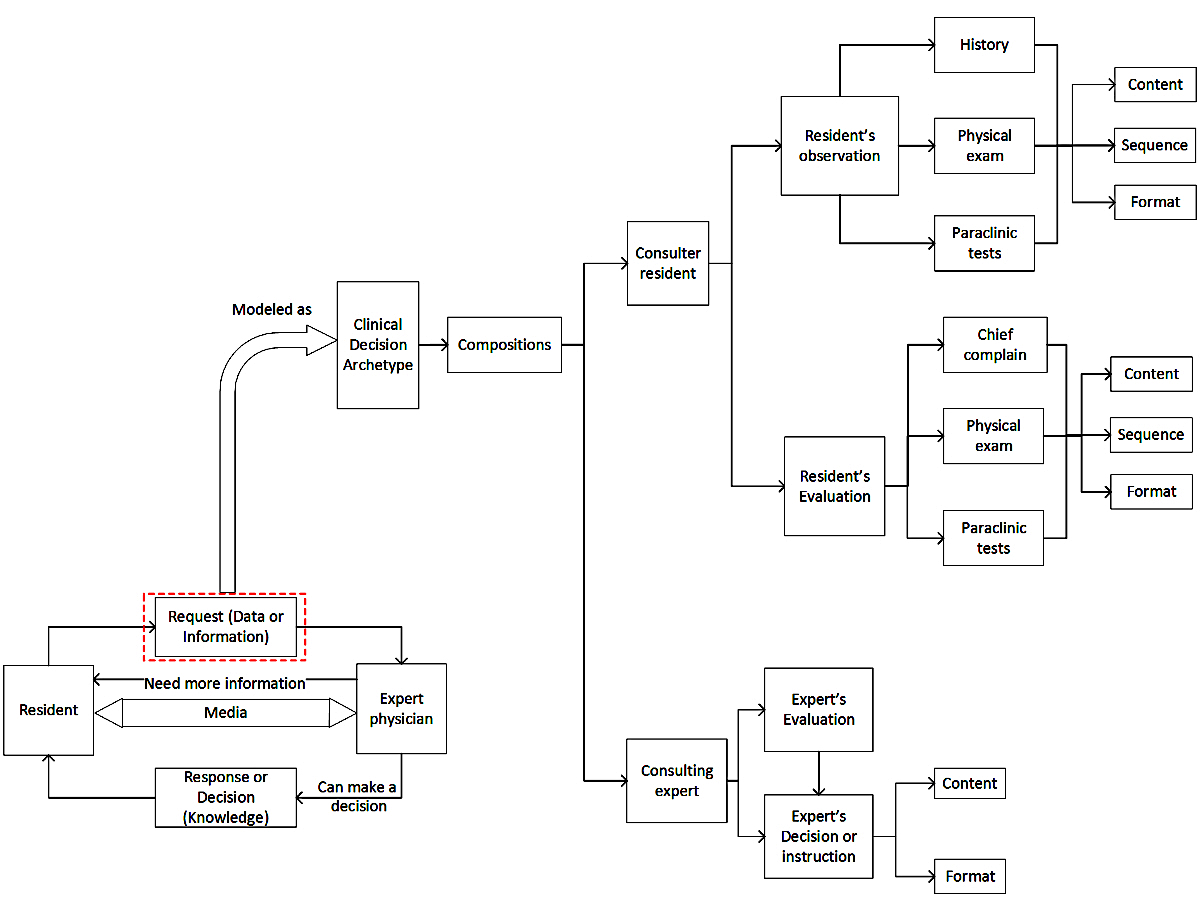
Designed model for teleconsultation, based on clinical decision-making archetype.

The result of our previous review study has shown that overall the teleconsultation model is almost identical among physicians and follows a general pattern of requesting consulting and sending patient information by the first physician and responding to it by the consulting physician. In this study, this overall model and pattern was used as the basis. However, in the section related to the exchange of data and information, the clinical archetypes particular to decision making were used (Figure 1).

As seen in Figure 1, the designed clinical archetypes consisted of two compositions associated with the request of the resident and the physician’s answer. Patient data and information collected by residents were grouped into two general categories of “observation” and “analysis.” Information related to the patient’s medical record, physical examination, and laboratory test results was inserted into the residents’ observation group.

**Figure 2:**
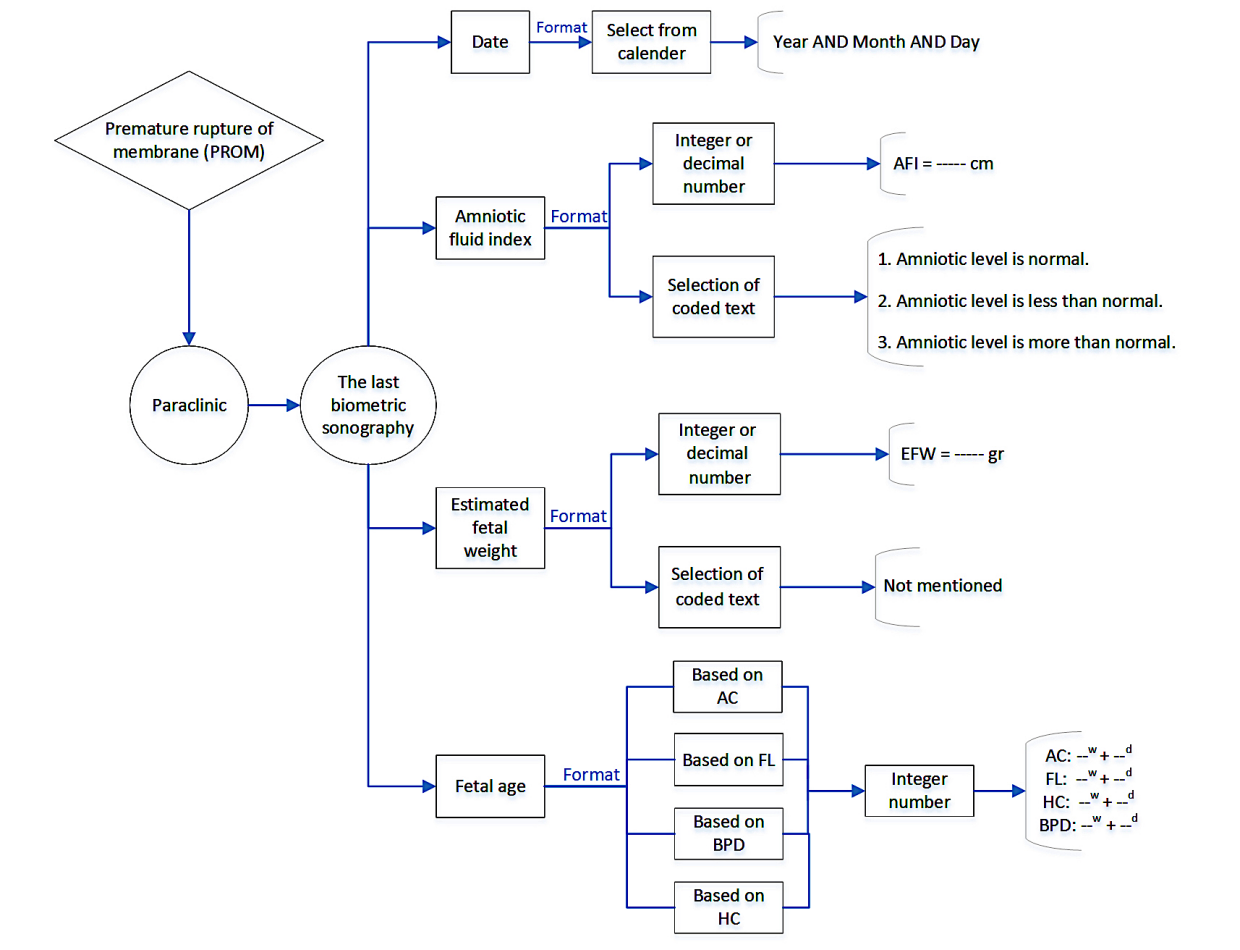
One of the subsets of designed model for premature rupture of membrane (PROM; the last biometric ultrasound). AFI: amniotic fluid index; EFW: estimated fetal weight; AC: abdominal circumference; FL: femur length; BPD: biparietal diameter; HC: head circumference.

These observations, especially those related to physical examinations and laboratory tests, were analyzed by the residents; in consultations between the senior residents and specialists, usually the resident’s analysis of such information was transferred to the specialist. For example, if the pregnant woman’s platelet count was 127,000, instead of the aforementioned number, the resident transferred their own analysis and impressions of the number as “normal platelet count” to the specialist. It should be noted that the residents were able to properly analyze only some of these cases, and in the case of other data and information, they had to use the correct analysis based on clinical experience of the on-call physician. The most important issue that should be considered in this archetype is determining a template for type and amount of information to be transferred to the specialist. As was also identified in the qualitative part of this project (and similar studies), experts believe that factors such as the personality of residents, patient presentation, and the amount of oral information received during the consultation have a great impact on decisions made by physicians. Hence, in this model, we were trying to extract the most common, necessary items used to make decisions about high-risk pregnancy by the review of the literature and surveys from local experts. Then, with the help of clinicians and informatics experts, proper format and report priority was set for all of these items’ components. In the next step, a structured format to provide a summary report of the completed items by the residents and to send it to the experts was designed. A subsidiary of one of the components of the designed model (the last available biometric ultrasound) is shown in more detail in Figure 2.

### Phase 2

#### The Result of the Checklist Design Step

At this point, a checklist that contained seven items was used for evaluation of the model (Multimedia Appendix 1).

#### The Result of Comparison of Archetype-Based Scenario With Routine-Based Scenario Group

The following table shows quartiles scores of the self-assessment checklist of specialists, divided into the archetype-based scenario and routine-based scenario groups (Table 1).

**Table 1:** Quartiles scores of self-assessment checklist of specialists in both groups.

Item number and description		Group	25th	Median	75th	*P* value
1	The quality of patient-related data and information provided on this consultation is acceptable.	AS^a^	4	4	5	<.001
		RS^b^	3	4	4	
2	The volume of information presented in this consultation, to decide about the patient, is enough.	AS	4	5	5	<.001
		RS	3	4	5	
3	There is additional nonuseful information in those provided about the patient.	AS	4	4	5	<.001
		RS	3	4	4	
4	Order and organization of the information in this consultation were acceptable.	AS	4	4	5	<.001
		RS	3	4	5	
5	According to the provided information, I’m sure about the decision for the patient.	AS	4	5	5	<.001
		RS	3	4	5	
6	The time required to make decisions with respect to the quality of information provided was acceptable.	AS	4	4	5	.122
		RS	3	4	5	
7	According to information provided, making the decision for the patient was easy for me.	AS	4	5	5	<.001
		RS	2.25	4	5	

^a^AS: archetype-based scenario.

^b^RS: routine-based scenario.

According to the results of the Wilcoxon test, in six items of the specialists’ self-assessment checklist, there was a significant difference between the scores assigned to the archetype-based scenario and those of the routine-based scenario groups, and in all of them (except the third item), the archetype-based scenario group received better scores than the other group. In the third item related to the presence of additional information in the scenarios, scores of the archetype-based scenario group were higher but worse than the routine-based scenario group. The only item that did not have a significant difference was the one related to the time required for clinical decision making of specialists.

## Discussion

### Principal Findings

In this study, to evaluate the proposed data model for teleconsultation archetype-based clinical decision-making consultation scenarios, we compared them with those scenarios based on routine procedures prepared by senior residents using the self-assessment checklist of obstetrics and gynecology physicians to rank the quality of their clinical decisions. The results of this comparison indicated that in five items (out of seven items in the checklist), scenarios based on archetypes received more favorable scores. This means that according to specialists, scenarios based on archetypes were better than scenarios based on routine procedures in terms of “quality of data,” “adequacy of information,” “discipline and organization,” “certainty of decision,” and “ease of decision making.” Although the average score of “acceptable time required for decision making” in the archetype-based scenario group was also higher than the routine-based scenario group, there was no statistically significant difference here. In the comparison between the two scenarios, the only item that experts evaluated unfavorable about scenarios based on archetypes was additional information in this scenario.

Results of previous studies have shown that increasing the quality of data and information exchange can improve the quality of decision making [13,14], although this increase depends on the quality of the individual who makes the decision. In other words, increasing the quality of data could increase the quality of decision making in the event that the decision-maker has knowledge about the relationship between the variables of the problem [14]. In this study, items related to acceptability of the quality of data and information in scenarios based on archetype received the higher score. The quality of sanitary data in some studies has been defined by two factors: accuracy and completeness [15]. It seems that evaluation of the accuracy of the data by specialists for any of the scenarios was not possible. Perhaps one of the reasons that specialists gave a higher score to this item in the archetype-based scenario compared with the routine-based scenario consultations was the completeness of this group’s scenarios. Due to the scenarios prepared by routine methods being mostly short, and on the assumption that it is not necessary to provide the specialist with some of this information during telephone consultation, residents removed them from their consultations, whereas in the interviews with specialists, they narrated experiences when inadequate information submitted by residents had jeopardized the life of the mother and the baby. Normally, if specialists need more information about the patient, they receive this information through frequent questions and answers with residents. Due to the absence of this approach at the time of writing of the routine scenarios by the resident, such scenarios might be imperfect. It is probable that the use of archetypes needed for clinical decision making improves different data quality and information factors, especially when there is no possibility of frequent questions and answers.

Another self-assessment checklist item was the adequacy of the information for clinical decision making. This item is also one of the aspects needed to enhance the quality of the decision [16,17]. As most managers tend to get as much information as possible, providing the right amount of information to them is a challenging task [16]. Hence, it is better to have a mechanism for determining the volume of incoming information to specialists to prevent information overload and ensure the adequacy of the information required, after all the delivery of clinical information more than the amount required typically does not improve consultation outcomes [18]. By definition, clinical archetypes can be used as a model to determine the structure and content needed to obtain clinical information [19]. They are also an appropriate and good way to describe structured sanitary information [20]. Thus, it is possible to build up the content needed for medical consultation by using a certain type of archetype for clinical tele–decision making. This could be approved according to the higher scores given by clinicians to the adequacy of the information item in the clinical archetype-based scenario in comparison with the routine-based scenario.

Bergus (2006) showed a significant correlation between the way the questions of physicians requesting consultation was organized and that of responses of consulting physicians. In fact, physicians requesting consultations can affect the outcome of consultation with specialists by how they design the questions and structure them; and this impact is independent of the specialists’ personal characteristics, the level of training, and the amount of information that is offered by the requesting consultations [18]. In a teledermatology study, a semistructured form was used to send information to physicians. However, the intended structure was only to determine the topic of the data entry fields, and text input was done freely. The most important declared finding of this study was the reduction in the number of patients referred to physicians, but it is not clear whether the use of this basic structure of the information sending form affected this reduction or not [21]. In another study, a pre-consultation structured questionnaire made students pay more attention to details of patient information [22]. Taken together, these studies demonstrate the positive results of using a specified structure and format in consultation. That is probably why the score associated with the acceptability of order and information structure item in archetype-based scenario (due to the specific structural for the display of data and their order of displaying) was higher than that of routine-based scenario.

In some studies, lack of request for conventional (in person) consultation is considered as an indicator to measure the physician’s confidence in teleconsultation, and in some other studies by using the 5-item Likert scale, the physician's confidence in clinical tele–decision making was measured. In the mentioned study, the confidence in decision making has been defined as a component consisting of data quality (such as quality of digital images) and the accuracy and details of the exchanged information. As a result, when digital image quality or resolution of radiological images was poor or amount of sent data was insufficient, the degree of confidence in the decision was reduced. The author of this study believes that sending more details, responding to the questions of consulting physicians, and increasing the number and quality of images could improve the confidence of surgeons to diagnose and treat patients [23]. It has been shown in other studies that the use of more advanced techniques of telemedicine has increased the confidence in the adequacy of the treatment and care provided by physicians and nurses [24]. In this study, specialists could ask more questions about the patients for consulting scenarios if more information is required. Information provided was inadequate, and thus, they felt a sense of uncertainty in the decision. However, only a small number of specialists have raised such questions at the end of scenarios (which could be because of the lack of questions for that scenario or because of the impatience of respondents), although the number of questions in routine-based scenario was more than those in the archetype-based scenario. Higher decision confidence scores may be explained by the above reason.

People usually tend to assess the quality of expectations and their feelings about the decisions that they have taken. One of the most basic and most important experiences after decision making is how comfortable the person feels about the decision taken. A comfortable decision is a decision that accompanies the sense of physical and mental ease and pleasure [25]. According to some studies, specialists believe that if clinical consultations and their related questions have a good structure, they could respond to them more easily than to unstructured questions [18]. In other words, it may be concluded that the structure and certain order in the archetype-based scenario can help the specialist to have a greater sense of comfort and ease when they are using such scenarios in comparison with the time they respond to the routine-based scenario.

The average scores of the item associated with the time needed to decide were the only average value that showed no significant difference between the two groups. In different studies, factors such as the low number of choices, fewer information inputs, and the limitation of the analysis, the small number of decision-makers and the low number of conflicting opinions are mentioned as factors that could increase the speed of decision making. Some other researchers believe that the greater the volume of information, the slower the decision making [26]. However, in our study, it seems that one of the factors that may result in higher average scores of “acceptability of the time required for decision making” item in the archetype-based scenario is sufficient volume of information needed for decision making, and of course, the order in presenting information to specialists.

In another study, in which the result were controversial, it was claimed that the accuracy of a diagnosis was directly associated with the processing speed of the information required to make decisions. In that study, it was concluded that increasing the speed of decision making (and reducing the time required for taking decisions) would increase the accuracy of diagnosis in physicians. In other words, spending a long time to diagnosis did not reduce the error rate in deciding [27]. Therefore, it is probably to expect if, with the improvement of various factors, the speed of physicians in decision making is accelerated, more accurate and less wrong decisions would be taken. Of course, the personality of the decision-maker should not be ignored. In interviews with specialists, it was also noted that the decision-making procedure and speed is different and this is because of the skills, previous experience in dealing with a variety of difficult clinical conditions, as well as the specific individual characteristics of physicians. Perhaps for this reason, there were no significant differences between the average scores of the time of decision making of the two groups. In other words, only modifications of external factors such as how to provide a consulting scenario cannot improve the speed of decision making because the internal and other important factors influence the process.

The only item where the archetype-based scenario received worse scores than the routine-based scenario was “the existence of additional unhelpful information” in these scenarios. As already mentioned, on the one hand, most decision-makers tend to gather as much information as possible to make better decisions [16], and on the other hand, it is better to improve the quality of decision making by a reduction in the volume of data inputs. Given that in the previous items medical specialists gave a higher score to the sufficient volume of archetype-based scenario in contrast to routine-based scenario, the existence of additional unhelpful information might be caused by items that normally are not mentioned because of their negative answer in routine consultation. For example, negative history for liver or kidney disease or absence of family history of hypertension are such information that in the specialists’ view, including written and electronics sources, are important to make decisions about diagnosis and treatment of pregnancy hypertension. Nonetheless, it seems these items along with their negative response, despite their importance in diagnosis and treatment, are interpreted as additional information. Most of our participants (specialists) believed that the lack of these items in the context of consultation meant that a particular item is negative, whereas in the world of information, the lack of these items, in addition to being a negative response, could be a sign that shows that the item is missed or left incomplete. So, ways to avoid misinterpretation in this regard should be looked for, and perhaps using techniques such as aggregation or summarization to reduce the amount of information available in the scenarios [16].

### Conclusions

Improving the quality of clinical care is partly associated with the improvement of decisions and judgments of the medical staff [28]. Our study has demonstrated that archetype-based consultation scenarios for clinical decision making were superior to routine-based scenarios in terms of quality, volume, and structure, and specialists felt more confident and comfortable using the archetype-based scenario for decision making. In addition, in terms of time, speed of decision making in these scenarios is estimated somewhat more acceptable than other scenarios. Nevertheless, there was futile and additional information in these scenarios, and by different techniques of aggregation and summarization, the substance of these scenarios can be improved. Along these lines, it appears that the proposed data model for archetype-based teleconsultation can enhance the quality and the nature of teleconsultation between physicians.

## Appendix

Multimedia Appendix 1 Self-assessment checklist of specialists for teleconsultations.

## Abbreviations

AS: archetype-based scenario
MUMS: Mashhad University of Medical Sciences
RS: routine-based scenario
SMS: short message service
