# Supplementary material for: A Data Model for Teleconsultation in Managing High-Risk Pregnancies: Design and Preliminary Evaluation
Source: JMIR Med Inform. 2017 Dec 14;5(4):e52. doi: 10.2196/medinform.8393 (PMC5746617; doi:10.2196/medinform.8393)
Supplement: Multimedia Appendix 1 [file medinform_v5i4e52_app1.pdf]

Appendix 1: Self-assessment checklist of specialists for teleconsultations

**Item No.**	**Item description**
1	The quality of patient-related data and information provided on this consultation is acceptable.
2	The volume of information presented in this consultation, to decide about the patient, is enough.
3	There is additional non-useful information in those provided about the patient.
4	Order and organization of the information in this consultation were acceptable.
5	According to the provided information, I'm sure about the decision for the patient.
6	The time required to make decisions with respect to the quality of information provided was acceptable.
7	According to information provided, making the decision for the patient was easy for me.
